# Consumers’ green purchase intention to visit green hotels: A value-belief-norm theory perspective

**DOI:** 10.3389/fpsyg.2023.1139116

**Published:** 2023-03-01

**Authors:** Cen-Peng Wang, Qi Zhang, Philip Pong Weng Wong, Lei Wang

**Affiliations:** ^1^School of Business, City University of Macau, Macao, Macao SAR, China; ^2^Business School, Faculty of Hospitality and Tourism, Xuzhou University of Technology, Xuzhou, Jiangsu, China; ^3^School of Hospitality, Sunway University, Bandar Sunway, Malaysia

**Keywords:** value-belief-norm theory, altruism, social norm, personal norm, green purchase implicit and explicit environmental attitude, intention to visit green hotels

## Abstract

**Introduction:**

The theory of planned behavior (TPB) has dominated the limited number of green hotel visitation studies; however, those studies’ findings are often inconclusive or even controversial. Thus, research needs to move beyond using the TPB to measure consumers’ intention and behavior, and to experiment with alternative theoretical frameworks to explain behavioral change. Value-belief-norm theory of environmentalism (VBN) proposed that various facets of values can influence individuals’ beliefs, subsequently effecting their moral obligations, ultimately, their pro-environmental behaviors. Hence, this study aims to examine the relationship between value components (i.e., biospheric, altruistic, collectivistic), beliefs (i.e., explicit and implicit attitude), norms (i.e., social and personal norm), and green purchase intention to visit green hotels.

**Methods:**

An online survey of convenience sampling technique was adopted for data collection. A total of 373 valid questionnaires were subjected to descriptive analysis, and confirmatory factor analysis and structural equation modeling were performed for the testing of the hypotheses.

**Results:**

The results suggested that biospheric and collectivistic value positively influence explicit environmental attitude while altruistic value positively influences intrinsic environmental attitude, but negatively influences extrinsic environmental attitude. Social norm was shown to have a positive impact on personal norm and green purchase intention. Furthermore, implicit environmental attitude was shown to influence personal norm and intention, while personal norm positively influences green purchase intention to visit green hotels.

**Discussion:**

This study provided an alternative perspective on the selection of green hotels among consumers based on value-belief-norm theory in the tourism literature. These empirical findings would greatly benefit green hotel managers and other key stakeholders in the hospitality industry.

## 1. Introduction

As a result of continuing economic growth, there has been an increasing trend of consumers’ focus on sustainable consumption over the past two decades (Wang et al., 2023). Environmental problems such as climate change, air pollution, waste generation, ozone depletion, and haze (Wang et al., 2022c) have attracted the attention of many different stakeholders such as business organizations, consumer groups, academic researchers, and governments (Kumar, 2021). Due to the increase in awareness of their consumption-related environmental problems, consumers are constantly seeking to purchase pro-environmental products or services for the benefit of future generations (Sheraz et al., 2021), and they are even willing to pay more for such products or services (D’Souza et al., 2020).

As the hospitality industry consume a great deal of energy and resources that negatively impact the environment (Ahmad et al., 2021), hotels need to implement green strategies to maintain competitiveness in the hospitality marketplace (Wang et al., 2020a), as they are able to charge higher rates to consumers who are willing to pay more to stay at green hotels (Patwary et al., 2022). According to Green Hotel Association (2023), green hotel refers to “environmentally-friendly properties whose managers are eager to institute programs that save water and energy and reduce generation of solid waste – while saving money – to help protect our one and only planet earth.” Over the last few decades, green hotels have become increasingly popular in some countries (Saleem, 2021). For example, even though the concept of green hotels have only been introduced in China recently from western countries (Wang and Wong, 2021), there are now more than 700 green hotels in China, with that number rapidly increasing (Wang, 2022). However, many scholars demonstrated that the booking revenues for green hotels has not increased significantly with the increase in the number of such establishments (D’Souza et al., 2020; Wang, 2022). In other words, although consumers claim that they are willing to visit green hotels, their behavior does not actually reflect that claim (Sadiq et al., 2022).

Nimri et al. (2020) stated that in the growing importance of pro-environmentally marketing, research has focused mainly on general environmental behavior rather than investigating consumers’ purchase patterns regarding specific products or service. Such studies may indeed provide some insights of pro-environmental behaviors, yet it might be challenging to generalize these findings to other green products or services (e.g., green hotel selection) (Wang et al., 2019). As such, there is little evidence to support the argument that consumer environmental awareness and beliefs can translate into green hotel selection (Wang et al., 2020a) and this phenomenon is generally known as the consumers’ green attitude-behavior gap (Wang, 2022).

The theory of reasoned action (TRA) and theory of planned behavior (TPB) have dominated the limited number of green hotel selection studies (Ulker-Demirel and Ciftci, 2020), but findings of such studies are often inconclusive or even controversial (Wang, 2022). Both theories are expectancy-value models which focus on rational reasoning, and they assume that self-interests and an individual’s weighted expected cost and benefits of alternatives (e.g., time, money, peer influence) are the main motivational factors for a person’s behavior (Wang et al., 2022a) and hence, they exclude some of the personal decision criteria (Ulker-Demirel and Ciftci, 2020). Consumers are motivated to endorse a green hotel primarily for the realization that their purchase decision plays a part in leaving a greener environment for future generations (Xu et al., 2022) and visiting green hotels can also be necessitated from having an emotional fondness for nature (Wang et al., 2023). Hence, consumers’ personal values, beliefs and norms should be taken into consideration when examining the factors that affect consumers’ green hotel selection (Wang et al., 2020a).

The value-belief-norm theory of environmentalism (VBN) is an alternative emerging theory applied by academics to explore and understand consumer pro-environmental behavior (Özekici, 2022). It is based on an individual’s ability to perform pro-environmental behavior without focusing on costs (Wang et al., 2022a), and it states that the more strongly individuals subscribe to values beyond their immediate own interests, that is, self-transcendent (i.e., altruistic or biospheric value) compared to self-enhancement (i.e., egoistic value), the more likely they are to engage in pro-environmental behavior (Rahman and Reynolds, 2019). Some studies have successfully predicted consumer pro-environmental behavior based on VBN (Han et al., 2019; Wang et al., 2022c) and indicated that VBN appeared to be successful in explaining low-cost environmental behavior and intention when compared to TPB (Wang et al., 2022a). However, research based on the application of VBN in environmental studies, such as green hotel visitation have been limited (Rahman and Reynolds, 2019).

Indeed, VBN states that altruistic, biospheric, and egoistic/collectivistic value that influence individuals’ beliefs components, will subsequently impact personal norms, which ultimately leads to pro-environmental behavior (Özekici, 2022). However, most previous studies on pro-environmental behavior have not differentiated biospheric value from altruistic value (Wang et al., 2022c). Even though the influence of egoistic value on consumer behavior is well substantiated, the validity of the measurement of egoistic value may not be appropriate in all settings, especially in highly collectivistic societies (Wang et al., 2022c).

Furthermore, the new environmental paradigm (NEP) reflects an individual’s ecological worldview that plays the central role in VBN (Wang et al., 2023), which directly affect one’s beliefs that environmental conditions threaten things that he values and he can act to reduce the threat (Stern, 2000). However, individuals’ ecological worldviews only reflect one’s concerns about the society and its problems that are related to the protection of the environment, thus named explicit attitude (Wang et al., 2023) but certain positive/negative attitudes can also be expressed through engaging in particular actual pro-environmental behaviors (Nimri et al., 2020) which refers to implicit attitude (Wang et al., 2023). There are very few studies that seeks to pursue a deeper understanding of the aspect of individual’s ecological worldview (i.e., explicit attitude and implicit attitude) that influence consumers’ green hotel selection (Patwary et al., 2022).

In addition, it is widely agreed in the literature that a high level of personal norm has a significant influence on consumer pro-environmental behavior (Arun et al., 2021), and VBN also proposed personal norm is the last important antecedent of pro-environmental behavior (Wang et al., 2022b). However, personal norm is only an internalized norm which reflects individuals’ personal feelings of moral obligations and ignores an individual’s value system that is influenced by social pressure which refers to social norms (Wang et al., 2023).

Moreover, research has shown that developing countries’ (e.g., China, India) consumers concerns and understanding of green hotels are still at a low level (Sadiq et al., 2022; Wang, 2022), whereby practice-related problems have persisted (Wang and Wong, 2021). One possible reason is that most previous studies related to pro-environmental behavior are written from the perspectives of western researchers (Arun et al., 2021) resulting in green hotel selection in those countries is still in its preliminary stage, lacking a unified definition and a systematic framework (Wang and Zhang, 2021).

Therefore, this study seeks to contribute to the research literature by examining: (1) the influence of biospheric value, altruistic value and collectivistic value on explicit environmental attitude and implicit environmental attitude respectively; (2) the influence of explicit environmental attitude and implicit environmental attitude on consumers’ personal norm and intention respectively; (3) the influence of social norm on explicit environmental attitude, implicit environmental attitude, personal norm and intention; and (4) the influence of explicit environmental attitude, implicit environmental attitude, and personal norm on intention to visit green hotels based on VBN in China. The results of this study can provide an alternative theoretical perspective on how consumers’ values, beliefs, and norms influence one’s intention to visit green hotels. The findings will also benefit researchers and green hotels’ operators in developing countries (e.g., China and India) to understand potential green hotel guests’ performances.

## 2. Literature review

### 2.1. The underpinning theory

**Figure 1:**
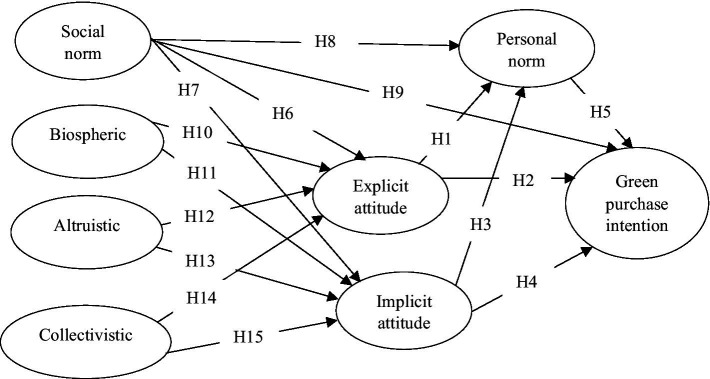
Theoretical research model.

This study proposes a theoretical research model which is based on VBN theory (see Figure 1). This theory is an extension of the moral norm-activation theory of altruism (NAM) (Schwartz, 1977) as NAM did not distinguish between the different types of pro-environmental behavior, resulting in the insufficient explanation for a variety of pro-environmental behavior indicators of non-activist environmentalism (Stern, 2000). Accordingly, values (i.e., altruistic, biospheric, and egoistic) influence individuals’ beliefs component involving their ecological worldview, in which individuals believe their behaviors can produce a good/bad outcome for the environment or for other individuals (Özekici, 2022). Subsequently, their beliefs are reflected in their pro-environmental personal norm, related to their sense of obligation to take certain actions, and ultimately, personal norm creates a general predisposition that influence all kinds of pro-environmental behavior (Wang et al., 2020a). Researchers demonstrated that VBN models displayed excellent predictive power in explaining low-cost pro-environmental behavior and high intention toward such behaviors when compared with TPB model (Steg and Vlek, 2009; Wang et al., 2022a).

### 2.2. Consumers’ green purchase intention to visit green hotels

Intention refers to the likelihood that an individual will engage in a given behavior (Sheraz et al., 2021), and his motivation to utilize the effort to perform the behavior (Nimri et al., 2020). In the same vein, green purchase intention is an individual’s commitment to the possibility and willingness of participating in pro-environmental behavior, e.g., visit green hotels (Eid et al., 2021) and intention is considered the best predictor of behavior (Saleem, 2021). Besides being a predictor of pro-environmental behavior, green purchase intention is also found to mediate the relationship between behavior and attitude, social norm, perceived behavioral control, and other antecedent variables (Sultana et al., 2022; Wibowo et al., 2022).

### 2.3. Explicit environmental attitude toward personal norm and intention

Explicit environmental attitude refers to the involvement of an individual with the society’s problems, particularly those related to environmental protection (Rahman and Reynolds, 2019). The central factors of personality and belief structure related to an individual’s ecological worldview is reflected in the NEP (Özekici, 2022). Similar to TPB, which posits that environmental behavioral beliefs shape attitude toward behavior (Rahman and Reynolds, 2016), and individuals’ ecological worldviews (i.e., NEP) affect their beliefs’ consequences whereby they believe that particular conditions pose threats to others and that there are certain actions they could initiate to avert those outcomes (Wang et al., 2022a). The NEP is most often used to measure ecological worldview (Milfont and Duckitt, 2010), which is also known as explicit environmental attitude (Wang et al., 2023). Individuals who scored high in NEP values (Levine and Strube, 2012) with an explicit environmental attitude will show high interest in social, political, and other issues relating to the protection of the natural environment, and will offer various possible solutions for those issues (Patwary et al., 2022). Individuals with an explicit environmental attitude can also exhibit different types of pro-environmental behavior that can have a direct effect on public policy-making, such as being a member of an environmental group, but also have an indirect effect on the natural environment, like developing new policies on environmental preservation (Leonidou et al., 2010). The NEP which is expressed by explicit environmental attitude can influence them in green hotel selection Sarabia-Andreu and Sarabia-Sánchez (2018) and Wang et al. (2023) found that explicit environmental attitude positively influenced consumers’ pro-environmental purchase behaviors. Hence, the following hypotheses are proposed:

*H1*: There is a positive relationship between explicit environmental attitude and personal norm.

*H2*: There is a positive relationship between explicit environmental attitude and intention.

### 2.4. Implicit environmental attitude toward personal norm and intention

Explicit environmental attitude is primarily dependent on individuals’ self-report measures of their attitudes (Sarabia-Andreu and Sarabia-Sánchez, 2018). By using a single NEP scale, explicit environmental attitude is readily accessible, self-reportable, and easily measured by standard questions, but is susceptible to bias related to social pressure and normative beliefs (Wang et al., 2023). A study by Corral-Verdugo (1997) indicated that there was a low correspondence between consumers’ self-reported attitude and observed re-use and recycling behavior. In contrast, intrinsic environmental attitude considers humans with other life forms as important, but only to the degree they influence humans or are of utility to humans (Rahman and Reynolds, 2019). It exists outside of conscious awareness and control and can influence behavior independently of explicit environmental attitude (Wang et al., 2023). In other words, it is the immediate reaction that one has to a stimulus and represent the unconscious awareness of such a reaction that makes it difficult to articulate feelings toward this stimulus (Greenwald and Banaji, 1995). Thus, intrinsic environmental attitude is the view that nature must be preserved for its internal values (Kortenkamp and Moore, 2001), and can be considered as behavioral beliefs that are included in TPB, which emphasize the importance of attitudes toward behavior (Patwary et al., 2022). Therefore, intrinsic environmental attitude becomes evident when the root of such attitude stems from an inherent care toward humankind (Rahman and Reynolds, 2019), and can be assessed through the strength of its associations with one’s positive or negative evaluative attributes with certain objects (e.g., green hotels) (Wang et al., 2023). Individuals adopting an implicit environmental attitude are more likely to engage in certain actual pro-environmental behavior (Sarabia-Andreu and Sarabia-Sánchez, 2018), such as green cars purchase (Wang et al., 2022c), and green hotels selection (Wang et al., 2020c). Hence, the following hypotheses are proposed:

*H3*: There is a positive relationship between implicit environmental attitude and personal norm.

*H4*: There is a positive relationship between implicit environmental attitude and intention.

### 2.5. Personal norm toward green purchase intention

The VBN shows that individuals’ personal norm is the key predictor of pro-environmental behavior, and it is influenced by values and beliefs simultaneously (Wang et al., 2022b). It reflects an individual’s sense of obligation to take certain pro-environmental actions (Fauzi et al., 2022) and is highly dependent on an individual’s feelings of his/her obligations that are related to one’s expectations (Sia and Jose, 2019). The concept of personal norm focuses solely on the evaluation of practices based on their moral self-worth (Han and Hyun, 2018) and previous studies have shown that personal norm is the single proximal determinant of pro-environmental behavior when compared to social norm (Saleem et al., 2021; Wang et al., 2022b). Hence, the suggested hypothesis is:

*H5*: There is a positive relationship between personal norm and intention.

### 2.6. Social norm toward personal norm, attitude, and green purchase intention

Although TPB proposed social norm is an important predictor of pro-environmental intention (Ajzen, 1991), the inconsistent predictive capacity of social norm revealed in past empirical studies has limited its usefulness as a predictor (Wang et al., 2019; Wang and Wong, 2021). In contrast with personal norm, which is an internalized norm which reflects individuals’ personal feelings of moral obligations (Han et al., 2019), social norm functions as a social pressure factor and inspires individuals on whether to perform or not to perform a behavior at a diverse macro-level setting (Wang et al., 2021). This is because an individual’s social norm influences his/her decision-making based on the perceived opinions of significant others (e.g., close friends, relatives, business partners, or co-workers/colleagues) (Wang and Wong, 2021) and is reflected in social costs and benefits (Wang et al., 2022c). Specifically, the significant others’ views on recycling performance can influence one’s personal feelings, moral duty or accountability in performing pro-environmental behavior (Wang et al., 2022b), and social norm has been found to positively affect personal norm toward pro-environmental behavior (Han et al., 2019). If an individual has a positive social norm toward green hotels, he/she will be more likely to have a favorable attitude to visit green hotels (Wang et al., 2019). Hence, the hypotheses developed for testing are as following:

*H6*: There is a positive relationship between social norm and explicit environmental attitude.

*H7*: There is a positive relationship between social norm and implicit environmental attitude.

*H8*: There is a positive relationship between social norm and personal norm.

*H9*: There is a positive relationship between social norm and intention.

### 2.7. Biospheric value toward attitude

The influence of biospheric value on pro-environmental behavior has not been resolved empirically as the concept of biospheric value is an emerging topic of study (Wang et al., 2020a), and most previous studies have not distinguished biospheric value from altruistic value (Wang et al., 2022c). For example, Gu (2022) reported that altruism which incorporated altruistic and biospheric values positively influence consumers pro-environmental behavior. However, some researchers believe that altruistic value and biospheric value should be tested separately (Rahman and Reynolds, 2016). Individuals who possess a high biospheric value will lend support to the protection of endangered animals, plants and habitats (Wang et al., 2020a) and biospheric value provides a distinct basis for people’s support for preserving the environment (Rahman and Reynolds, 2016). In contrast, the role of altruistic value reflects the individuals’ concerns about the welfare of others (Wang et al., 2022c). Hence, some researchers have demonstrated that biospheric value is the most important principle leading to pro-environmental behavior and it explains the process of forming ecological conscious attitude better than altruistic value (Rahman and Reynolds, 2016; Wang et al., 2020a). Hence, the following hypotheses are proposed:

*H10*: There is a positive relationship between biospheric value and explicit environmental attitude.

*H11*: There is a positive relationship between biospheric value and implicit environmental attitude.

### 2.8. Altruistic value toward attitude

Altruistic value is professed by individuals who are concerned for the welfare of society and others (Gu, 2022). It represents the act of doing something good for others without expecting anything in return (Rahman and Reynolds, 2019). In green marketing, altruistic value is a personal value structure which leads to pro-environmental behavior (Wang et al., 2020a). However, there is insufficient research on the relationship between altruistic value and green hotel visitation in the literature, and little is known about the role of altruistic value’s influence on green hotel selection (Wang et al., 2020a). For hotel consumers, the major motivator for them to visit green hotels is the feeling that their purchasing behavior can help save the planet and leave a better environment for future generations (Teng et al., 2015). Eid et al. (2021) reported that altruistic value positively influences consumers’ patronage attitudes and intention of visiting a green hotel, and Wang et al. (2022c) reported similar results in China. Thus, the following hypothesis is proposed:

*H12*: There is a positive relationship between altruistic value and explicit environmental attitude.

*H13*: There is a positive relationship between altruistic value and implicit environmental attitude.

### 2.9. Collectivistic (egoistic) value toward attitude

The egoistic or individualistic value is the third value component of beliefs toward pro-environmental behavior in the VBN model. It refers to individuals who focus on maximizing their outcomes based on self-interest (Rahman and Reynolds, 2016). Accordingly, individualistic value tends to be independent and self-oriented (Kim and Choi, 2005). There are two types of individuals who have individualistic value orientation: (1) individuals with a strong selfish and competitive orientation and are less likely to practice pro-environmental behavior; and (2) individuals who have satisfied their own needs who are more likely to act ecologically as they consume more resources and thus tend to care more about bigger social and pro-environmental issues (Wang et al., 2020a). However, collectivistic values can be considered a more important predictor of pro-environmental attitudes as collectivistic people tends to be more cooperative, more willing to help others, and emphasize group goals compared with individualistic people (Wang et al., 2022c). This type of attitude is evident in highly collectivistic societies such as China, along with those in Japan and Korea (Wang et al., 2020a).

Individuals with collectivistic values are more interdependent and group-oriented, and they may forego individual motivations for that which is good for the group and others (Handique, 2014). Thus, egoistic value and collectivistic value are two opposite values (Wang et al., 2020a). The influence of these two major values of individualistic and collectivistic value on consumer pro-environmental behavior has been demonstrated in a previous study by Wang et al. (2020a). Specifically, researchers indicated that there is a positive relationship between collectivistic value and pro-environmental behavior (Kim and Choi, 2005) and consumers from collectivistic countries and nature oriented cultures, such as the Chinese are even more likely to be green (Coleman et al., 2011). Chen (2013) reported that there is a significant difference between the collectivistic value (Chinese) and individualistic value (American) consumers with respect to environmental attitudes, knowledge, social norm and intention, while collectivistic value can explain a high percentage of the variation in consumer pro-environmental attitude and intention (Kumar and Sreen, 2020; Wang et al., 2020a). Thus, the following hypotheses are established:

*H14*: There is a positive relationship between collectivistic value and explicit environmental attitude.

*H15*: There is a positive relationship between collectivistic value and implicit environmental attitude.

## 3. Research methodology

### 3.1. Data collection

The non-probability sampling method was used as it was not possible to acquire accurate information relating to sampling frames for this type of study. A convenience sampling technique was used to collect data due to its advantages such as geographical proximity, easy accessibility, availability at a given time or the willingness to participate, for the purpose of the study (Etikan et al., 2016). An online/web-based sampling was utilized for data collection as there were more than 1,011 million regular internet users in China at the June 2021, and the internet penetration has reached 71.6% among the Chinese population (China Internet Network Information Center, 2021). In addition, the online/web-based sampling method has some advantages when compared with traditional sampling methods, such as instant access to a wider audience; high speed of data collection; low cost; better access to unique populations; and data can be collected irrespective of the geographic location of audience (Evans and Mathur, 2005; Wright, 2005). In addition, many past studies related to pro-environmental behavior/green hotel selection had adopted an online/web-based sampling method to collect data with satisfactory results (Wang et al., 2019; Wang and Wong, 2021).

The research questionnaires were posted on the electronic collection website, named,^1^ from 1 June to 31 August. This free online survey questionnaire collection website is the most popular among businesses, organizations, and consumers in China, and it is frequently sued to collect primary survey data from internet users (Wang and Wong, 2021). For a variety of research surveys, gifts or money are usually offered as an incentive to increase the response rate (Wang et al., 2019). Therefore, an online red envelope (i.e., money of 5 RMB/per respondent) is offered as an incentive to invite internet users to complete the questionnaire on this website. Respondents need to complete all questionnaires items in sequence that reflect the significant elements of the study and demographic questions were placed toward the end of the questionnaire. The questionnaire items were translated into Chinese *via* the back-translation method, which means that the questionnaire was initially translated into Chinese and then back translated to English by a second team of translators. A pilot test was conducted involving 30 respondents to ensure the usability and validity of the developed instrument and prevent any issues that may affect the quality of the obtained data. The obtained sample size for the pilot test in this study exceeded the recommendation for a pilot test in most studies, which is 10% of the target sample size for the actual data collection (Connelly, 2008; Hertzog, 2008). Furthermore, the content validity of the questionnaire was evaluated by asking several experts from India, Malaysia and China to ensure adequate coverage of the investigative questions and a few questionnaires were also distributed to some undergraduate students to detect face validity issues. Finally, there are no incomplete/invalid questionnaires received and a total of 373 questionnaires were returned for analysis. For structural equation modeling (SEM) a minimum sample size of 200 respondents and between 10 and 20 cases per parameter (Tabachnick and Fidell, 2012; Kline, 2015) is recommended. There were 36 items (per parameter) in the questionnaires, and the 373 responses received exceeded the suggested minimal sample size.

### 3.2. Measurement

The research instrument for this study is a self-administered questionnaire, with close-ended questions which allows for quicker collection time and reduced levels of bias (Saunders et al., 2011). All items in the questionnaire were validated and adapted from previous published studies, using the seven-point Likert scale, from (1) strongly disagree to (7) strongly agree. A seven-point Likert scale is more likely to produce slightly higher mean scores within the highest possible attainable scores and can enable easier comparisons of data (Dawes, 2008). The questionnaire was designed in five sections: The first section included items for measuring the values variables: biospheric, altruistic, and collectivistic. Six items measured the biospheric value; another six items were used to assess altruistic value; and a further six items evaluated the collectivistic value, and these items were adopted from Wang et al. (2020a). The second section includes questions to establish explicit and implicit attitude; with four items belonging to explicit attitude and another four items were used to measure implicit attitude which were adapted from Leonidou et al. (2010). The third section of questionnaire was to evaluate the variables of social norm and personal norm. Three items that were used to measure social norm were adapted from Han and Yoon (2015), and three items assessing personal norm were adapted from Chen and Tung (2014) and Han and Hyun (2018). The fourth section includes questions to establish green purchase intention, with four items adapted from Wang et al. (2020b). The last section includes questions on demographic characteristics such as age, gender, educational level, and income level.

## 4. Data analysis and results

### 4.1. Descriptive analysis

Of the 373 respondents, 34.6% of the respondents were in the age group of 18–30, 52.5% were male, while 31.9% of respondents had completed a four-years bachelor’s degree. The monthly income of most of respondents (31.4%) were in the income group of 3,001–4,500 Chinese yuan (RMB) (see Table 1). Distributed data is normal when skewness and kurtosis values are nearer to zero. Skewness that ranges from −2 to +2, and kurtosis that ranges from −7 to +7 exhibits a stronger deviation from normality (Byrne, 2016). The results showed that that data distribution is normal as skewness were between −1.6 to −0.622, while kurtosis ranged from −1.306 to +2.981. Besides, the multivariate normality has been checked by using Mahalanobis distance. The results showed that the Mahalanobis distance maximum value was 13.448 which is less than the critical chi-square value of 15.51, *p* < 0.05, preserving multivariate normality. The Kaiser-Meyer-Olkin (KMO) and Bartlett’s test of sphericity showed sampling adequacy with 0.801, *p* < 0.001 value.

**Table 1:** Sample characteristics (*N* = 373).

	Characteristic	Frequency	Percentage (%)
Gender	Male	196	52.5
	Female	177	47.5
Age	Below 18	63	16.9
	18–30	129	34.6
	31–45	97	26
	46–60	61	16.3
	Above 61	23	6.2
Education level	Middle school	26	7
	High school	55	14.7
	Diploma	114	30.6
	Bachelor	119	31.9
	Master and above	59	15.8
Income level	Below 1700	47	12.6
	1701–3,000	103	27.6
	3,001–4,500	117	31.4
	4,501–6,000	54	14.5
	Above 6,001	52	13.9

### 4.2. Confirmatory factor analysis

Factor loading shows the variance explained by the variable on that particular factor. According to Hair et al. (2010), the rule of thumb for assessing the practical significance of a factor is that the factor loading must be at least 0.5; ideally 0.7 or higher. A cut-off value of factor loadings of 0.6 was set for achieving a better outcome in this study. Thus, items of factor loadings of below 0.6 were dropped, which included the following items: collectivistic6; explicit attitude1; and intention1.

In the model fit summary, the model Chi-square is 1599.481, df = 447, *p* < 0.001. Chi-square divided by the df value (CMIN/DF) = 3.578, meets the suggested guideline of <5.0 (Bentler, 1990). The Parsimony Goodness of Fit Index (PGFI) of 0.617 meets the threshold value of >0.5 (Mulaik et al., 1989). The Comparative Fit Index (CFI) of 0.909 exceeded the suggested guideline of >0.9 (Bentler, 1990). The Parsimony Normed Fit Index (PNFI) = 0.691, Parsimonious Comparative Fit Index (PCFI) = 0.727, Incremental Fit Index (IFI) = 0.91, RMR = 0.078, and the Root Mean Square Error of Approximation (RMSEA) = 0.097, all meeting the minimum requirements of PNFI and PCFI >0.5; IFI > 0.9; RMSEA <0.08, where between 0.8–1 is moderate fit (Ho, 2006; Meyers et al., 2006). There should be at least three indices to be met to ensure model fit (Ho, 2006), hence, model fit of the measurement model was achieved.

Furthermore, a calculation of composite reliability (CR) and validity revealed a range of CR from 0.754 to 0.925 meeting Hair et al. (2010)’s threshold for reliability, i.e., CR should be >0.7. For convergent validity, the average variance extracted (AVE) should be greater than 0.5 (Fornell and Larcker, 1981), and the results showed AVE values ranges from 0.506 to 0.752. For discriminant validity to exist, both maximum shared squared variance (MSV) and the average shared squared variance (ASV) should be less than the AVE (Hair et al., 2010) and the correlation between different variables must be less than 0.9 (Meyers et al., 2006). Having met these required thresholds, the reliability (see Table 2) and validity (see Table 3) of the measurement model were established.

**Table 2:** Reliability and convergent validity of measurement model.

**Variables (Cronbach’s Alpha)**x	**Items**	**Item loadings**	**CR**	**AVE**	**S.D.**
Biospheric (*α* = 0.932)	1. Respecting the earth.	0.797	0.924	0.669	1.17
	2. Unity with nature.	0.863			1.141
	3. Protecting the environment.	0.778			1.21
	4. Preventing pollution.	0.806			1.11
	5. Visit at green hotel helps conserve natural resources.	0.819			1.095
	6. Visit at green hotel helps decrease pollution.	0.843			1.232
Altruistic (α = 0.937)	1. I have given directions to stranger.	0.823	0.925	0.674	1.292
	2. I have given money or donated goods to a charity or group.	0.96			1.289
	3. I have given money to a stranger who needed it.	0.855			1.2
	4. I have pointed out a clerk’s error.	0.706			1.387
	5. I have let a neighbor whom I did not know too well borrow an item of some value to me.	0.822			1.427
	6. I have offered my seat on a bus or train to a stranger who was standings.	0.735			1.283
Collectivistic (α = 0.928)	1. I like to help others in the time of need.	0.907	0.92	0.7	1.24
	2. I like to maintain ward relationships with others.	0.713			1.197
	3. To do well in life, the help of friends is crucial.	0.894			1.21
	4. One of the pleasures in life is to be interdependently related to others.	0.76			1.087
	5. One of the pleasures of life is to feel part of a large group of people.	0.891			1.253
Social norm (α = 0.806)	1. Most people who are important to me think I should stay at a green hotel when traveling.	0.751	0.808	0.583	1.302
	2. Most people who are important to me would want me to stay at a green hotel when traveling.	0.783			1.329
	3. People whose opinion I value would refer me stay at a green hotel when traveling.	0.757			1.24
Personal norm (α = 0.748)	1. I feel an obligation to treasure natural resources by choosing green hotels when traveling.	0.646	0.754	0.506	1.2
	2. I feel an obligation to save natural resources because they are limited by choosing green hotels when traveling.	0.723			1.17
	3. I feel an obligation to act pro-environmentally by choosing green hotels when traveling.	0.761			1.373
Explicit attitude (α = 0.915)	2. Anti-pollution laws should be enforced more strongly.	0.795	0.901	0.752	1.236
	3. Major social changes are necessary to protect the natural environment.	0.85			1.185
	4. Humans are severely abusing the environment.	0.95			1.197
Implicit attitude (α = 0.9)	1. I am very concerned about the environment.	0.828	0.9	0.694	1.005
	2. I would be willing to reduce my consumption to help protect the environment.	0.875			0.984
	3. I would give part of my own money to help protect wild animals.	0.789			0.961
	4. I have asked my family to recycle some of the things we use.	0.837			1.016
Intention (α = 0.804)	2. I will make an effort to stay at a green hotel when traveling.	0.738	0.806	0.581	1.103
	3. I am likely to stay in a hotel implementing environmental strategies.	0.794			1.187
	4. I am more likely to stay in a green hotel over a non-green hotel.	0.753			1.193

**Table 3:** Correlation between variables and discriminate validity.

**Items**	1	2	3	4	5	6	7	8	AVE	MSV	ASV
1. Intention	**0.762**								0.581	0.407	0.089
2. Altruistic	0.094	**0.821**							0.674	0.391	0.154
3. Biospheric	0.093	0.621	**0.818**						0.669	0.507	0.171
4. Collectivistic	−0.008	0.625	0.533	**0.837**					0.7	0.391	0.133
5. Social norm	0.638	0.16	0.066	0.07	**0.764**				0.583	0.407	0.071
6. Personal norm	0.397	0.165	0.066	0.097	0.232	**0.712**			0.506	0.158	0.044
7. Explicit attitude	0.04	0.455	0.712	0.481	0.009	−0.017	**0.867**		0.752	0.507	0.135
8. Implicit attitude	0.202	0.177	0.068	0.117	0.035	0.239	−0.005	**0.833**	0.694	0.057	0.021

Bold values are denote the square root of AVE.

### 4.3. Structural equation modeling

**Figure 2:**
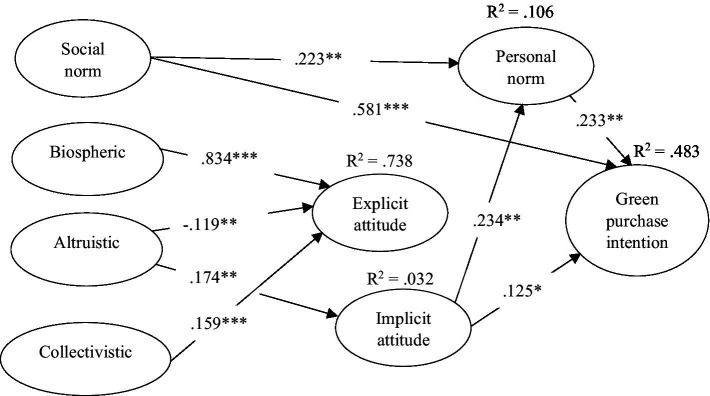
The structural model results. *p < 0.05, **p < 0.01, ***p < 0.001, Critical ratio (C.R.) > 1.96.

After performing a maximum likelihood estimation method, the model fit of the structural model showed a good outcome. The overall goodness-of-fit indices of the structural model met the required thresholds as follows: Chi-square is 1638.85, df = 447, p < 0.001, CMIN/DF = 3.666, PGFI = 0.609, CFI = 0.904, IFI = 0.906, PNFI = 0.687, PCFI = 0.723, and RMSEA = 0.098. The next step was to perform SEM to test the hypotheses developed for the study and the results are shown in Table 4 and Figure 2.

**Table 4:** Structural relationships and hypotheses testing.

Hypothesis	Parameter	β	*p*-value	C.R.	S.E.	Decision
H1	Explicit attitude → Personal norm	−0.004	0.958	−0.053	0.052	Rejected
H2	Explicit attitude → Intention	0.045	0.435	0.78	0.045	Rejected
H3	Implicit attitude → Personal norm	0.234	0.001	3.179	0.067	Supported
H4	Implicit attitude → Intention	0.125	0.043	2.201	0.059	Supported
H5	Personal norm → Intention	0.233	0.001	3.193	0.077	Supported
H6	Social norm → Explicit attitude	−0.044	0.292	−1.06	0.045	Rejected
H7	Social norm → Implicit attitude	0.013	0.857	0.18	0.061	Rejected
H8	Social norm → Personal norm	0.223	0.004	2.862	0.062	Supported
H9	Social norm → Intention	0.581	***	7.254	0.066	Supported
H10	Biospheric → Explicit attitude	0.834	***	13.49	0.073	Supported
H11	Biospheric → Implicit attitude	−0.027	0.675	−0.42	0.062	Rejected
H12	Altruistic → Explicit attitude	−0.119	0.003	−2.99	0.04	Rejected
H13	Altruistic → Implicit attitude	0.174	0.008	2.664	0.053	Supported
H14	Collectivistic → Explicit attitude	0.159	***	3.989	0.036	Supported
H15	Collectivistic → Implicit attitude	0.023	0.722	0.355	0.048	Rejected

## 5. Discussion and conclusion

The current study examined: (1) the relationship between the value components (biospheric value, altruistic value, and collectivistic value) and belief components of ecological worldview (explicit attitude) and implicit attitude; (2) the relationship between explicit/implicit attitude and pro-environmental personal norms; (3) how personal norms influence consumers’ green purchase intention; and (4) the effect of social norms on explicit attitude, implicit attitude, personal norms and intentions toward green hotel selection. According to Wang et al. (2022a), individuals’ ecological worldview (i.e., explicit environmental attitude) has a direct impact on their personal norms, which will affect their pro-environmental intention/behavior (Rahman and Reynolds, 2019). However, the results of this study demonstrated that explicit attitude did not influence individuals’ personal norm (β = −0.004, *p* > 0.05) and intention (β = 0.45, *p* > 0.05) to visit green hotels. In contrast, individuals’ positive evaluation (i.e., implicit environmental attitude) of green hotels’ attributions significantly influenced their personal norm (β = 0.234, *p* < 0.05) and intention (β = 0.125, *p* < 0.05) to visit green hotels. This corresponds with the findings of some researchers who argue that there is a positive significant relationship between an individual’s attitude toward a certain object (e.g., green hotels) and intention to visit that (Wang et al., 2020a; Özekici, 2022). Thus, H1 and H2 were rejected, and H3 and H4 were supported.

Our results showed that individuals who have a strong sense of obligation to take pro-environmental actions will possess a higher personal norm toward visiting green hotels (β = 0.233, *p* < 0.05). This corresponds with some previous studies have shown that personal norm is broadly used as the most proximal determinant of pro-environmental behavior (Han and Hyun, 2018; Fauzi et al., 2022). Therefore, H5 is accepted. Previous studies showed that individuals’ perceived social pressure significantly influenced their explicit environmental attitude (Wang et al., 2022a) as well as implicit attitude (Wang et al., 2019, 2022b). Nevertheless, current study’s results showed that social norm did not influence either explicit attitude (β = −0.44, *p* > 0.05) or implicit attitude (β = 0.013, *p* > 0.05) toward green hotel selection. Thus, H6 and H7 were rejected. In addition, the findings of this study showed that social norm positively and significantly influenced one’s personal norm (β = 0.223, *p* < 0.05) and intention (β = 0.581, *p* < 0.05). Those findings corresponds to certain empirical studies that showed an individual’s perceived social pressure significantly influence his or her personal sense of moral obligation and intention to practice sustainable consumption (Liu et al., 2020; Wang et al., 2022b). Thus, H8 and H9 were accepted.

Furthermore, previous studies showed that value components (i.e., altruistic value, biospheric value, collectivistic value) positively influence individuals’ pro-environmental explicit attitude (Özekici, 2022; Wang et al., 2022a) or implicit attitude (Wang et al., 2020a, 2022c) in green marketing. The findings showed that the biospheric value and collectivistic value positively influenced explicit attitude, respectively, (β = 0.834, *p* < 0.05; β = 0.159, *p* < 0.05), and altruistic value negatively influenced explicit attitude since β = −0.119, *p* < 0.05. This means that China is a highly collectivistic value country, and individuals who come from collectivist countries are nature oriented cultures, and are more likely to go green (Coleman et al., 2011). Meanwhile, with the rapid development of the economy and improvement of living conditions, most Chinese citizens are starting to realize that China is the largest “world factory,” having a negative impact on the environment. This negative impact is especially evident in recent times when Chinese citizens are facing serious environmental issues of haze pollution and food safety. Another reason for going green is that the Chinese government is placing special emphasis on implementing its eco-friendly policies. Therefore, being concerned about the environmental welfare of others is less important than preserving the natural environment and moving forward with the same goal of protect the environment. Hence, H10 and H14 were supported and H12 was rejected.

However, when taking certain pro-environmental actions on a particular behavior (e.g., green hotels), concern about others’ welfare is more important than concern about natural environment and their common goals of protect the environment as only altruistic value positively influenced implicit attitude (β = 0.174, *p* < 0.05). This is line with past studies showing that altruistic value plays an important role in determining one’s pro-environmental attitude (Eid et al., 2021; Gu, 2022). Thus, H13 is accepted. However, our results showed that biospheric value and collectivistic value did not influence implicit attitude since β = −0.27, *p* > 0.05 and β = 0.023, *p* > 0.05, respectively. These findings are inconsistent with past studies showing that biospheric value and collectivistic value positively influenced one’s pro-environmental attitude toward a specific object (D’Souza et al., 2020; Saleem, 2021). Therefore, H11 and H15 are rejected.

### 5.1. Theoretical contributions

Researchers indicated that there is a significant difference between implicit and explicit environmental attitude regarding green purchase intention and behavior (Sarabia-Andreu and Sarabia-Sánchez, 2018; Wang et al., 2023). Explicit environmental attitude (i.e., NEP) plays a center role in determining consumers’ personal norm, which will affect their pro-environmental behavior in VBN (Özekici, 2022). Ideally, individuals have high scores on explicit environmental attitude based on NEP will show high interest in social, political and other issues relating to the protection of the natural environment, and will offer various opinions and suggestions to how to solve those issues (Leonidou et al., 2010). Some researchers criticized that consumers’ explicit environmental attitudes are based on self-report and measured by standard questions (Sarabia-Andreu and Sarabia-Sánchez, 2018), and they highlighted that self-reports are not completely reliable measures of one’s actual behavior (Wang et al., 2023). Current study’s results showed that explicit environmental attitude did not influence consumers’ personal norm and intention to visit green hotels. This in line with Sarabia-Andreu and Sarabia-Sánchez (2018) suggestion that an explicit environmental attitude plays a significant but not absolute role in explaining one’s decision-making and consequently, one’s intention to act.

In contrast, implicit environmental attitude seems to be more suitable for measuring one’s attitude toward a certain type of pro-environmental behavior (i.e., green hotels selection) (Levine and Strube, 2012; Wang et al., 2023). This study found that implicit environmental attitude positively influenced consumers’ personal norm and intention to visit green hotels. The results suggest that individuals who have high implicit environmental attitudes related to their strong sense of obligation to take pro-environmental actions will possess a higher personal norm toward visiting green hotels. In addition, this study finds low correlations between explicit and implicit environmental attitudes, and it appears to support the argument that they are different attitudinal constructs that refer to different mental processes (Sarabia-Andreu and Sarabia-Sánchez, 2018).

Social norm is the most complicated variable in TPB as previous empirical studies showed it had multiple correlations with one’s attitude (Wang and Wong, 2021) and personal norm (Wang et al., 2022b). On one hand, those findings are inconsistent with the original framework of TPB which postulated that attitude and social norm were independent of one another (Wang et al., 2019); on the other hand, the TPB has been criticized for its lack of consideration of personal norm on pro-environmental behavior (Liu et al., 2020). This research confirmed the active role of social norm in affecting personal norm and intention to visit green hotels. Social norm functions reflected in social costs and benefits tends to influence individuals’ self-judgments and feeling of moral obligations on whether to perform or not to perform a behavior. The findings showed that an individual’s close friends, relatives, co-workers or colleagues, even business partners who have positive attitudes or enjoyable experiences with green hotels, can share their positive opinions with each other, leading to a stronger sense of obligation and a higher possibility, to visit green hotels. The findings should be of special interest to academics from highly collectivistic societies because in these countries, consumers’ purchase formation was derived from others who are close to themselves, which is then translated to personal norm and intention. In the same vein, further investigation into the correlations between social norm and personal norm also can be added to TPB models to provide a more exhaustive explanation of consumers’ pro-environmental behaviors.

Furthermore, previous studies on green purchase behavior did not distinguish the difference between altruistic value and biospheric value (Wang et al., 2022c). Specifically, previous studies that showed the negative relationship between egoism and pro-environmental behavior might be less apparent in high collectivistic societies (e.g., China, India, and Korea) (Wang et al., 2022a). This study tested the relationship between three types of values (i.e., biospheric, altruistic, and collectivistic value) and explicit and implicit environmental attitude toward green hotel selection. Consumers’ biospheric and collectivistic value positively influenced their explicit environmental attitudes, but not implicit environmental attitudes. Chinese consumers’ general pro-environmental attitude (i.e., explicit environmental attitude) is derived from their concern about the planet, natural resources, and the pursuit of harmonious interrelationships within groups. This underpins the theory that individuals who come from collectivistic countries and nature oriented cultures, such as the Chinese, are more likely to go green (Coleman et al., 2011).

Meanwhile, results of this study confirmed that there is a positive relationship between altruistic value and consumers’ implicit environmental attitude of green hotels attributes. Individuals who are more concerned about the significant others are more willing to perform certain actual pro-environmental behavior that they perceived can produce a pro-environmental outcome for them. Nevertheless, results from this study showed that consumers expressed that they had negative perceptions toward explicit environmental attitude which may mean that they do not believe that they can protect the whole eco-system through their pro-environmental actions. Overall, this study suggested that biospheric, altruistic and collectivistic value seems to be more suitable in predicting consumers’ pro-environmental attitude in collectivistic societies or regions.

In addition, most of the previous studies related to green purchase attitude-behavior gap have adapted the TRA and TPB models to predict consumers green hotel selection (Wang et al., 2019) as both of these theories have dominated this particular field of research (Rahman and Reynolds, 2016). This study was among the first that empirically tested and validated the significant relationships of reformed value components (i.e., biospheric, altruistic, collectivistic), belief components (i.e., explicit and implicit environmental attitude), normative components (social norm and personal norm) and consumers’ intention to visit green hotels, using the highly rigorous method of SEM. The results showed that biospheric, altruistic, and collectivistic value significantly influenced the explicit and implicit environmental attitude, respectively. Furthermore, implicit environmental attitude exerted a positive influence on personal norm, ultimately resulting in intention to visit green hotels. These findings suggest that the reformed VBN model provides a more comprehensive explanation of consumers’ decision-making processes relating to green hotels visitation, especially significant for certain countries or regions with communities which profess a low degree of environmental awareness and knowledge about green hotels.

### 5.2. Management implications

Marketing to the Chinese market is quite different from marketing to western consumers since the Chinese culture is more collectivist in nature, similar to that of Japan and Korea (Wang and Wong, 2021). Collectivistic people tend to be more cooperative, more willing to help others, and emphasize group goals compared to individualistic people (Wang et al., 2020a). This study demonstrated that consumers’ biospheric value and collectivistic value are positively related to explicit environmental attitude, respectively. There was no significant relationship between biospheric and collectivistic value and implicit environmental attitude, while explicit environmental attitude was not significantly related to personal norm and green purchase intention. This means that with the rapid development of the economy and improvement of living conditions, Chinese citizens are at the same time, facing serious environmental issues, such as haze pollution, and over-utilization of natural resources (Wang, 2022). Hence, the Chinese government is placing special emphasis on implementing its eco-friendly policies to achieve all Chinese pro-environmental goals (Jiang and Gao, 2019). For green hotel operators, they should implement a publicity campaign on their green hotels’ pro-environmental objectives in line with the country’s expectations. Green practices such as saving water, electricity and reducing the usage of non-durable items should be highlighted in their promotional campaigns to potential consumers.

Green hotels operators should educate potential consumers what they can do when they are visiting a green hotel by promoting practices such as using recyclable pens, slippers, recycled toiletries containers and even encouraging consumers to use their own toiletries (e.g., toothbrush, toothpaste, towel). As an incentive, green hotels should provide a competitive rate for consumers who perform such ecological behaviors by offering a discount for consumers who take own toiletries when they are visiting a green hotel.

In addition, social norm plays an important role in influencing consumers’ personal norm and intention to visit green hotels, while personal norm had a positive influence on intention. This shows that Chinese individuals’ close-friends, relatives, colleagues, co-workers, or business counterparts help shape their moral obligation and intention to visit green hotels. Therefore, for hospitality operators who are interested in exploiting the intention of consumers to visit green hotels, they should learn how to advertise and promote their green hotels more effectively to the target public. Environmental promotion should be a part of green hotels’ marketing campaigns by highlighting the importance of pro-environmental strategies, and how such practices help to preserve the environment for future generations. For example, in green hotels’ advertisements, they can publicize their hotels’ savings in energy and water usage through adopting green strategies which can lead to low carbon emission. Green hotels’ operators should consider the importance of user-generated forums, word-of-mouth, and e-word-of-mouth as important channels of communication. They can publish certain pro-environmental strategies notifications about what they have implemented in online chat groups or their own websites/applications for members, thus, attracting potential consumers to spread such information among their friends.

Last but not least, consumers are visiting green hotels due to the concerns about the welfare of others and society. Therefore, governmental agencies working together with green hotels should promote pro-environmental business practices so that a positive image for the hospitality industry can be portrayed. Moreover, many studies showed that although consumers have expressed high inclinations to stay at green hotels during their travel (Arun et al., 2021; Rawashdeh and Al-Ababneh, 2021), revenues and bookings received by green hotels have remained stagnant (Wang and Wong, 2021). Therefore, the policy makers should introduce certain implementable strategies to stimulate the customers take-up rate, such as offering green subsidies for green hotel guests, employees, and the green hotel industry in general. This will incentivize potential consumers to patronize green hotels and the green hotels’ management to implement green business practices.

### 5.3. Limitations and future research

There were some limitations inherent in this study. The first limitation concerns the data collection; data was only collected *via* an online survey from a broad range of online Chinese netizens. Although the use of online surveys has become more popular, adopting this approach has several well-known disadvantages. For example, there is no guarantee that participants will provide accurate demographic or characteristic information (Wright, 2005). Also, the convenience sampling technique was used to collect data in this study. Although this sampling technique is used widely, it is prone to bias and influences that are beyond the researchers’ control, as some of the respondents might not be providing accurate answers either knowingly or unknowingly (Saunders et al., 2011). Therefore, the model used and results acquired from this study cannot be representative of the population and should be tested in other regions to further confirm its validity and generalizability. The second limitation is that some studies have shown that using demographic characteristics to predict consumer GPB can produce inconsistent and even contradictory results (Wang et al., 2020b). Lastly, intention is only one of the factors affecting behavior, and pro-environmental behavior is also influenced by other factors such as resources, opportunities, time, money, etc. (Paul et al., 2016). Therefore, further studies are needed to investigate the influence of such factors on consumers’ intention to visit green hotels.

## Data availability statement

The raw data supporting the conclusions of this article will be made available by the authors, without undue reservation.

## Author contributions

C-PW contributed to the drafting the manuscript, data collection, data interpretation, and final approval of the version to be published. QZ contributed to the data collection, data analysis, and data interpretation. PW contributed to the drafting the manuscript, critical revision of the manuscript, and final approval of the version to be published. LW contributed to the design of the work, data interpretation, drafting the manuscript, critical revision of the manuscript, and final approval of the version to be published. All authors contributed to the article and approved the submitted version.

## Conflict of interest

The authors declare that the research was conducted in the absence of any commercial or financial relationships that could be construed as a potential conflict of interest.

## Publisher’s note

All claims expressed in this article are solely those of the authors and do not necessarily represent those of their affiliated organizations, or those of the publisher, the editors and the reviewers. Any product that may be evaluated in this article, or claim that may be made by its manufacturer, is not guaranteed or endorsed by the publisher.

## Footnotes

^1^ http://www.wenjuan.com http://www.wenjuan.com

## References

[ref1] Ahmad Z. Hassan N. M. Khattak M. N. Moustafa M. A. Fakhri M. (2021). Impact of tourist's environmental awareness on pro-environmental behavior with the mediating effect of tourist's environmental concern and moderating effect of tourist's environmental attachment. Sustainability 13:12998. doi: 10.3390/su132312998

[ref2] Ajzen I. (1991). The theory of planned behavior. Organ. Behav. Hum. Decis. Process. 50, 179–211. doi: 10.1016/0749-5978(91)90020-T

[ref3] Arun T. M. Kaur P. Bresciani S. Dhir A. (2021). What drives the adoption and consumption of green hotel products and services? A systematic literature review of past achievement and future promises. Bus. Strateg. Environ. 30, 2637–2655. doi: 10.1002/bse.2768

[ref4] Bentler P. M. (1990). Comparative fit indexes in structural models. Psychol. Bull. 107, 238–246. doi: 10.1037/0033-2909.107.2.238 2320703

[ref5] Byrne B. M. (2016). Structural Equation Modeling with AMOS: Basic Concepts, Applications, and Programming. London: Routledge.

[ref6] Chen L. (2013). A study of green purchase intention comparing with collectivistic (Chinese) and individualistic (American) consumers in Shanghai. IMBR 5, 342–346. doi: 10.22610/imbr.v5i7.1061

[ref7] Chen M.-F. Tung P.-J. (2014). Developing an extended theory of planned behavior model to predict consumers’ intention to visit green hotels. Int. J. Hosp. Manag. 36, 221–230. doi: 10.1016/j.ijhm.2013.09.006

[ref8] China Internet Network Information Center. (2021). The 48th statistical report on China's internet development Available at: http://www.cnnic.com.cn/IDR/ReportDownloads/202111/P020211119394556095096.pdf (Accessed August 2, 2021).

[ref9] Coleman L. J. Bahnan N. Kelkar M. Curry N. (2011). Walking the walk: how the theory of reasoned action explains adult and student intentions to go green. J. Appl. Bus. Res. 27, 107–116. doi: 10.19030/jabr.v27i3.4217

[ref10] Connelly L. M. (2008). Pilot studies. Medsurg Nurs. 17, 411–413. doi: 10.12968/bjon.2008.17.7.29056 19248407

[ref11] Corral-Verdugo V. C. (1997). Dual ‘realities’ of conservation behavior: self-reports vs observations of re-use and recycling behavior. J. Environ. Psychol. 17, 135–145. doi: 10.1006/jevp.1997.0048

[ref12] D’Souza C. Apaolaza V. Hartmann P. Brouwer A. R. (2020). Marketing for sustainability: Travellers’ intentions to stay in green hotels. J. Vacat. Mark. 27, 187–202. doi: 10.1177/1356766720975063

[ref13] Dawes J. (2008). Do data characteristics change according to the number of scale points used? An experiment using 5-point, 7-point and 10-point scales. Int. J. Mark. Res. 50, 61–104. doi: 10.1177/147078530805000106

[ref14] Eid R. Agag G. Shehawy Y. M. (2021). Understanding guests’ intention to visit green hotels. J. Hospital. Tour. Res. 45, 494–528. doi: 10.1177/1096348020947800

[ref15] Etikan I. Musa S. A. Alkassim R. S. (2016). Comparison of convenience sampling and purposive sampling. Am. J. Theor. Appl. Stat. 5, 1–4. doi: 10.11648/j.ajtas.20160501.11

[ref16] Evans J. R. Mathur A. (2005). The value of online surveys. Internet Res. 15, 195–219. doi: 10.1108/10662240510590360

[ref17] Fauzi M. A. Hanafiah M. H. Kunjuraman V. (2022). Tourists' intention to visit green hotels: building on the theory of planned behaviour and the value-belief-norm theory. Journal of tourism. Futures, 1–22. doi: 10.1108/JTF-01-2022-0008

[ref18] Fornell C. Larcker D. F. (1981). Evaluating structural equation models with unobservable variables and measurement error. J. Mark. Res. 18, 39–50. doi: 10.2307/3151335

[ref19] Green Hotel Association. (2023). What are green hotels? Available at: http://www.greenhotels.com (Accessed January 4, 2023).

[ref20] Greenwald A. G. Banaji M. R. (1995). Implicit social cognition: attitudes, self-esteem, and stereotypes. Psychol. Rev. 102, 4–27. doi: 10.1037/0033-295x.102.1.4, PMID: 7878162

[ref21] Gu S. (2022). The effects of subjective knowledge, altruistic value and consumer self-confidence on the green purchase attitudes and green purchase behaviour of Chinese customers. Turyzm/Tourism 32, 7–27. doi: 10.18778/0867-5856.32.2.01

[ref22] Hair J. F. Black W. C. Babin B. J. Tatham R. L. (2010). Multivariate Data Analysis: A Global Perspective (7th Hoboken: Pearson Prentice Hall.

[ref23] Han H. Hwang J. Lee M. Kim J. (2019). Word-of-mouth, buying, and sacrifice intentions for eco-cruises: exploring the function of norm activation and value-attitude-behavior. Tour. Manag. 70, 430–443. doi: 10.1016/j.tourman.2018.09.006

[ref24] Han H. Hyun S. S. (2018). College youth travelers’ eco-purchase behavior and recycling activity while traveling: an examination of gender difference. J. Travel Tour. Mark. 35, 740–754. doi: 10.1080/10548408.2017.1405865

[ref25] Han H. Yoon H. J. (2015). Hotel customers’ environmentally responsible behavioral intention: impact of key constructs on decision in green consumerism. Int. J. Hosp. Manag. 45, 22–33. doi: 10.1016/j.ijhm.2014.11.004

[ref26] Handique K. (2014). le of collectivism, environmental concern, scepticism and perceived consumer effectiveness on green purchasing behaviour of consumers of Guwahati, India. Int. J. Bus. Manag. 2, 58–66.

[ref27] Hertzog M. A. (2008). Considerations in determining sample size for pilot studies. Res. Nurs. Health 31, 180–191. doi: 10.1002/nur.20247 18183564

[ref28] Ho R. (2006). Handbook of Univariate and Multivariate Data Analysis and Interpretation with SPSS. New York, NY: CRC Press.

[ref29] Jiang Y. Gao Y. (2019). Factors that influence potential green hotel customers’ decision-making process – evidence from China. J. China Tour. Res. 15, 455–477. doi: 10.1080/19388160.2018.1558139

[ref30] Kim Y. Choi S. M. (2005). Antecedents of green purchase behavior: an examination of collectivism, environmental concern, and PCE. Adv. Consum. Res. 32, 592–599. https://www.acrwebsite.org/volumes/9156/volumes/v32/NA-32

[ref31] Kline R. B. (2015). Principles and Practice of Structural Equation Modeling (4th). New York: Guilford.

[ref32] Kortenkamp K. V. Moore C. F. (2001). Ecocentrism and anthropocentrism: moral reasoning about ecogogical commons dilemmas. J. Environ. Psychol. 21, 261–272. doi: 10.1006/jevp.2001.0205

[ref33] Kumar G. A. (2021). Framing a model for green buying behavior of Indian consumers: from the lenses of the theory of planned behavior. J. Clean. Prod. 295:126487. doi: 10.1016/j.jclepro.2021.126487

[ref34] Kumar S. Sreen N. (2020). “Role of internal and external values on green purchase” in Green Marketing as a Positive Driver Toward Business Sustainability. eds. Naidoo V. Verma R. (Pennsylvania: IGI Global)

[ref35] Leonidou L. C. Leonidou C. N. Kvasova O. (2010). Antecedents and outcomes of consumer environmentally friendly attitudes and behaviour. J. Mark. Manag. 26, 1319–1344. doi: 10.1080/0267257X.2010.523710

[ref36] Levine D. S. Strube M. J. (2012). Environmental attitudes, knowledge, intentions and behaviors among college students. J. Soc. Psychol. 152, 308–326. doi: 10.1080/00224545.2011.604363 22558826

[ref37] Liu M. T. Liu Y. Mo Z. (2020). Moral norm is the key: an extension of the theory of planned behaviour (TPB) on Chinese consumers' green purchase intention. Asia Pac. J. Mark. Logist. 32, 1823–1841. doi: 10.1108/APJML-05-2019-0285

[ref38] Meyers L. S. Gamst G. Guarino A. J. (2006). Applied Multivariate Research: Design and Interpretation. London: Sage.

[ref39] Milfont T. L. Duckitt J. (2010). The environmental attitudes inventory: a valid and reliable measure to assess the structure of environmental attitudes. J. Environ. Psychol. 30, 80–94. doi: 10.1016/j.jenvp.2009.09.001

[ref40] Mulaik S. A. James L. R. Van Alstine J. Bennett N. Lind S. Stilwell C. D. (1989). Evaluation of goodness-of-fit indices for structural equation models. Psychol. Bull. 105, 430–445. doi: 10.1037//0033-2909.105.3.430

[ref41] Nimri R. Patiar A. Jin X. (2020). The determinants of consumers’ intention of purchasing green hotel accommodation: extending the theory of planned behaviour. J. Hosp. Tour. Manag. 45, 535–543. doi: 10.1016/j.jhtm.2020.10.013

[ref42] Nimri R. Patiar A. Kensbock S. Jin X. (2020). Consumers’ intention to stay in green hotels in Australia: theorization and implications. J. Hospital. Tour. Res. 44, 149–168. doi: 10.1177/1096348019862602

[ref43] Özekici Y. K. (2022). Extending value-belief and norm theory with social identity for preventing food waste at restaurants. Turizm Akademik Dergisi 9, 273–291.

[ref44] Patwary A. K. Rasoolimanesh S. M. Rabiul M. K. Aziz R. C. Hanafiah M. H. (2022). Linking environmental knowledge, environmental responsibility, altruism, and intention toward green hotels through ecocentric and anthropocentric attitudes. Int. J. Contemp. Hosp. Manag. 34, 4653–4673. doi: 10.1108/IJCHM-01-2022-0039

[ref45] Paul J. Modi A. Patel J. (2016). Predicting green product consumption using theory of planned behavior and reasoned action. J. Retail. Consum. Serv. 29, 123–134. doi: 10.1016/j.jretconser.2015.11.006

[ref46] Rahman I. Reynolds D. (2016). Predicting green hotel behavioral intentions using a theory of environmental commitment and sacrifice for the environment. Int. J. Hosp. Manag. 52, 107–116. doi: 10.1016/j.ijhm.2015.09.007

[ref47] Rahman I. Reynolds D. (2019). The influence of values and attitudes on green consumer behavior: a conceptual model of green hotel patronage. Int. J. Hosp. Tour. Adm. 20, 47–74. doi: 10.1080/15256480.2017.1359729

[ref48] Rawashdeh A. A. Al-Ababneh M. M. (2021). Hotel guests’ perceptions of environmentally friendly practices in Jordan. J. Environ. Manag. Tour. 12, 107–120. doi: 10.14505/jemt.v12.1(49).09

[ref49] Sadiq M. Adil M. Paul J. (2022). Eco-friendly hotel stay and environmental attitude: a value-attitude-behaviour perspective. Int. J. Hosp. Manag. 100:103094. doi: 10.1016/j.ijhm.2021.103094

[ref50] Saleem F. (2021). Antecedents of the green behavioral intentions of hotel guests: a developing country perspective. Sustainability 13:4427. doi: 10.3390/su13084427

[ref51] Saleem M. A. Eagle L. Low D. (2021). Determinants of eco-socially conscious consumer behavior toward alternative fuel vehicles. J. Consum. Mark. 38, 211–228. doi: 10.1108/JCM-05-2019-3208

[ref52] Sarabia-Andreu F. Sarabia-Sánchez F. J. (2018). Do implicit and explicit attitudes explain organic wine purchase intention? An attitudinal segmentation approach. Int. J. Wine Bus. Res. 30, 463–480. doi: 10.1108/IJWBR-09-2017-0063

[ref53] Saunders M. Lewis P. Thornhill A. (2011). Research Methods for Business Students (5th). Bengaluru: Pearson Education.

[ref54] Schwartz S. H. (1977). Normative influences on altruism. Adv. Exp. Soc. Psychol. 10, 221–279. doi: 10.1016/S0065-2601(08)60358-5

[ref55] Sheraz N. Saleem S. Sultan S. (2021). The consumer’s pro environmental attitude and its impact on green purchase behavior. J. Contemp. Issues Bus. Govern. 27, 221–233. doi: 10.47750/cibg.2021.27.05.015

[ref56] Sia S. K. Jose A. (2019). Attitude and subjective norm as personal moral obligation mediated predictors of intention to build eco-friendly house. Manag. Environ. Qual. Int. J. 30, 678–694. doi: 10.1108/MEQ-02-2019-0038

[ref57] Steg L. Vlek C. (2009). Encouraging pro-environmental behaviour: an integrative review and research agenda. J. Environ. Psychol. 29, 309–317. doi: 10.1016/j.jenvp.2008.10.004

[ref58] Stern P. C. (2000). New environmental theories: toward a coherent theory of environmentally significant behavior. J. Soc. Issues 56, 407–424. doi: 10.1111/0022-4537.00175

[ref59] Sultana N. Amin S. Islam A. (2022). Influence of perceived environmental knowledge and environmental concern on customers' green hotel visit intention: mediating role of green trust. Asia Pac. J. Bus. Admin. 14, 223–243. doi: 10.1108/APJBA-08-2021-0421

[ref60] Tabachnick B. G. Fidell L. S. (2012). Using Multivariate Statistics (6th). Boston: Allyn & Bacon/Pearson Education.

[ref61] Teng Y.-M. Wu K.-S. Liu H.-H. (2015). Integrating altruism and the theory of planned behavior to predict patronage intention of a green hotel. J. Hospital. Tour. Res. 39, 299–315. doi: 10.1177/1096348012471383

[ref62] Ulker-Demirel E. Ciftci G. (2020). A systematic literature review of the theory of planned behavior in tourism, leisure and hospitality management research. J. Hosp. Tour. Manag. 43, 209–219. doi: 10.1016/j.jhtm.2020.04.003

[ref63] Wang L. (2022). Determinants of consumers purchase attitude and intention toward green hotel selection. J. China Tour. Res. 18, 203–222. doi: 10.1080/19388160.2020.1816241

[ref64] Wang L. Shao Y.-X. Heng J.-Y. Cheng Y. Xu Y. Wang Z.-X.. (2023). A deeper understanding of attitude and norm applicable to green hotel selection. J. Qual. Assur. Hosp. Tour., 1–33. doi: 10.1080/1528008X.2023.2165594. [Epub ahead of print].

[ref65] Wang L. Wang Z.-X. Zhang Q. Jebbouri A. Wong P. P. W. (2022a). Consumers’ intention to visit green hotels – a goal-framing theory perspective. J. Sustain. Tour. 30, 1837–1857. doi: 10.1080/09669582.2021.1977937

[ref66] Wang L. Wong P. P. W. (2021). Marketing of environmentally friendly hotels in China through religious segmentation: a theory of planned behaviour approach. Tour. Rev. 76, 1164–1180. doi: 10.1108/TR-08-2019-0327

[ref67] Wang L. Wong P. P. W. Elangkovan N. A. (2020a). Antecedents of green purchase behaviour: an examination of altruism and environmental knowledge. Int. J. Cult. Tour. Hospital. Res. 14, 63–82. doi: 10.1108/IJCTHR-02-2019-0034

[ref68] Wang L. Wong P. P. W. Elangkovan N. A. (2020b). The demographic impact of consumer green purchase intention toward green hotel selection in China. Tour. Hosp. Res. 20, 210–222. doi: 10.1177/1467358419848129

[ref69] Wang L. Wong P. P. W. Elangkovan N. A. (2020c). The influence of religiosity on consumer’s green purchase intention towards green hotel selection in China. J. China Tour. Res. 16, 319–345. doi: 10.1080/19388160.2019.1637318

[ref70] Wang L. Wong P. P. W. Elangkovan N. A. Chee W. M. (2019). Green hotel selection of Chinese consumers: a planned behavior perspective. J. China Tour. Res. 15, 192–212. doi: 10.1080/19388160.2018.1553743

[ref71] Wang L. Wong P. P. W. Zhang Q. (2021). Travellers’ destination choice among university students in China amid COVID-19: extending the theory of planned behaviour. Tour. Rev. 76, 749–763. doi: 10.1108/TR-06-2020-0269

[ref72] Wang L. Zhang Q. (2021). The role of extrinsic religiosity on consumer green hotel selection in China. Int. J. Tour. Hotel Bus. Manag. 3, 405–425.

[ref73] Wang L. Zhang Q. Ding Y.-Y. Wong P. P. W. (2022b). The effect of social and personal norm on intention to patronize green hotels: extension of theory of planned behavior. J. China Tour. Res., 1–24. doi: 10.1080/19388160.2022.2070567. [Epub ahead of print].

[ref74] Wang L. Zhang Q. Wong P. P. W. (2022c). Purchase intention for green cars among Chinese millennials: merging the value–attitude–behavior theory and theory of planned behavior. Front. Psychol. 13:786292. doi: 10.3389/fpsyg.2022.786292, PMID: 35273539 PMC8902249

[ref75] Wibowo S. F. Najib M. Sumarwan U. Asnawi Y. H. (2022). Rational and moral considerations in organic coffee purchase intention: evidence from Indonesia. Economies 10:308. doi: 10.3390/economies10120308

[ref76] Wright K. B. (2005). Researching internet-based populations: advantages and disadvantages of online survey research, online questionnaire authoring software packages, and web survey services. J. Comput. Mediat. Commun. 10:259. doi: 10.1111/j.1083-6101.2005.tb00259.x

[ref77] Xu S. Zhou X. Ahmad Z. (2022). Measuring the psychological behavior of tourism service providers in low-income regions: implementing effective service marketing and performances strategies. Sustainability 14:11459. doi: 10.3390/su141811459

